# Association of Breast Cancer Screening Behaviors With Stage at Breast Cancer Diagnosis and Potential for Additive Multi-Cancer Detection *via* Liquid Biopsy Screening: A Claims-Based Study

**DOI:** 10.3389/fonc.2021.688455

**Published:** 2021-06-15

**Authors:** Christine Hathaway, Peter Paetsch, Yali Li, Jincao Wu, Sam Asgarian, Alex Parker, Alley Welsh, Patricia Deverka, Ariella Cohain

**Affiliations:** ^1^ Thrive, An Exact Sciences Company, Cambridge, MA, United States; ^2^ Blue Health Intelligence, Chicago, IL, United States; ^3^ Deverka Consulting, LLC, Apex, NC, United States

**Keywords:** liquid biopsy, cancer screening, breast cancer, preventive care, mammography, earlier detection, claims data analysis, multi-cancer early detection

## Abstract

**Purpose:**

To evaluate mammography uptake and subsequent breast cancer diagnoses, as well as the prospect of additive cancer detection *via* a liquid biopsy multi-cancer early detection (MCED) screening test during a routine preventive care exam (PCE).

**Methods:**

Patients with incident breast cancer were identified from five years of longitudinal Blue Health Intelligence^®^ (BHI^®^) claims data (2014-19) and their screening mammogram and PCE utilization were characterized. Ordinal logistic regression analyses were performed to identify the association of a biennial screening mammogram with stage at diagnosis. Additional screening opportunities for breast cancer during a PCE within two years before diagnosis were identified, and the method extrapolated to all cancers, including those without recommended screening modalities.

**Results:**

Claims for biennial screening mammograms and the time from screening to diagnosis were found to be predictors of breast cancer stage at diagnosis. When compared to women who received a screening mammogram proximal to their breast cancer diagnosis (0-4 months), women who were adherent to guidelines but had a longer time window from their screening mammogram to diagnosis (4-24 months) had a 87% increased odds of a later-stage (stages III or IV) breast cancer diagnosis (p-value <0.001), while women with no biennial screening mammogram had a 155% increased odds of a later-stage breast cancer diagnosis (p-value <0.001). This highlights the importance of screening in the earlier detection of breast cancer. Of incident breast cancer cases, 23% had no evidence of a screening mammogram in the two years before diagnosis. However, 49% of these women had a PCE within that time. Thus, an additional 11% of breast cancer cases could have been screened if a MCED test had been available during a PCE. Additionally, MCED tests have the potential to target up to 58% of the top 5 cancers that are the leading causes of cancer death currently without a USPSTF recommended screening modality (prostate, pancreatic, liver, lymphoma, and ovarian cancer).

**Conclusion:**

The study used claims data to demonstrate the association of cancer screening with cancer stage at diagnosis and demonstrates the unmet potential for a MCED screening test which could be ordered during a PCE.

## Introduction

In 2021, there are expected to be approximately 1.9 million new cancer cases and over 600,000 cancer deaths reported in the United States, making it the second leading cause of death in the country (1). Stage at diagnosis is a key predictor of cancer prognosis. For example, the 5-year survival rate for breast cancer is 99% for early stage, localized diagnoses but 28% for later-stage, distant diagnoses (2). Similarly, the 5-year survival rate for colorectal cancer is 90% for localized and 14% for distant (2). Survival varies by cancer type, but treatment of earlier stage cancer is consistently associated with improved survival relative to treatment of later stage cancers. This suggests that earlier detection of cancer is critical to improving patient health outcomes.

The United States Preventive Services Task Force (USPSTF) evidence review of the benefits of screening for breast cancer revealed that mammography reduced breast cancer mortality by 7.7 deaths per 10,000 women screened for those aged 50-59 and by 21.3 deaths per 10,000 women screened for those aged 60-69 (3). A similar USPSTF evidence review of colorectal cancer screening methods demonstrated a significant reduction in cancer-specific mortality with use of flexible sigmoidoscopy, fecal occult blood test, or colonoscopy compared to no screening (4). However, only four cancer types have a USPSTF recommendation for screening (breast, cervical, colorectal, and lung).

Even the cancer types that do have a USPSTF recommendation for screening still have gaps in adherence due to a variety of barriers and factors that affect access and uptake. National Health Interview Survey (NHIS) results for 2015 showed self-reported rates of adherence with Pap smears (cervical cancer), mammograms (breast cancer), and colorectal cancer screening tests were 81%, 71%, and 63% respectively, which were all lower than the Healthy People 2020 targets of 93%, 81%, and 70% for these three cancers (5). Additionally, lung cancer screening, which up to March 2021 was recommended for those with a 30 pack-year smoking history, only had a 13% adherence rate (6).

Longitudinal claims data are useful for documenting use of procedures, irrespective of the care setting, and for examining the temporal relationship between procedures and clinical events. Importantly, researchers have developed and validated effective means of using claims data to classify cancers by stage, expanding the utility of claims data for cancer health services research (7–11). Claims data are also useful in documenting patterns of routine care, which may reveal opportunities for intervention such as counseling patients regarding healthy behaviors and the importance of cancer screening. Despite compelling evidence for the survival benefits of screening for breast, colorectal, lung and cervical cancers, there is a paucity of claims-based studies investigating the relationship between screening and cancer stage at diagnosis, while also quantifying the potential additive impact of novel screening technologies when incorporated into routine care.

Recent studies evaluating the use of liquid biopsy tests (also referred to as “blood-based tests”) to detect multiple cancer types in asymptomatic persons demonstrate their promise as effective screening tools (12–14). These tests have the opportunity to shift the paradigm from one screening test detecting one cancer to one screening test detecting multiple cancers. Additionally, the use of blood-based screening tests may support earlier detection of cancer types for which no guideline approved screening method currently exists, as well as identify cancers missed by extant screening methods. The simplicity and potential accessibility of a blood-based test, delivered at a routine physical or even in the local community or home, could also help reduce some of the barriers to screening as a whole, potentially enabling broader and more equitable preventive care. It is anticipated that blood-based tests for multi-cancer screening will be available for clinical adoption within the next 2 years (15).

An annual wellness or preventive care exam (PCE) is a visit that often includes age and gender-appropriate history-taking, counseling on risk factor reduction, and ordering of diagnostic procedures including routine bloodwork (e.g. lipid and metabolic panels, blood counts). These preventive visits also allow providers to note abnormal physical findings or symptoms that could indicate a need for further diagnostic evaluation. PCEs also represent a potential opportunity for providers to recommend a blood-based cancer screening test. Understanding PCE utilization in combination with an existing screening method such as mammography provides insight into the added opportunity of a blood-based screening test. To date, the patterns of routine preventive care utilization in an insured population have not been evaluated in relation to subsequent incident cancer diagnoses.

To address this, we utilized five years of longitudinal data from the Blue Health Intelligence^®^ (BHI^®^) national database. BHI is an independent data and analytics company that is a licensee of the Blue Cross and Blue Shield Association; BHI data are sourced from a large number of health insurance plans. Using these data we sought to evaluate the association between screening test utilization and cancer stage at diagnosis. We selected breast cancer incidence in women eligible for biennial screening mammograms as our target condition, as the available claims data set spanned five years and we wished to capture complete screening intervals. Other cancers such as lung cancer or colorectal cancer were not suitable for this study, owing to the absence of patient-reported data such as smoking history in claims data, or to guideline-recommended screening intervals that exceeded our time horizon (e.g. colonoscopy screening every ten years). Cervical cancer was also not included in the analysis due to very low incidence of this cancer during the available five years of claims data.

**Figure 1:**
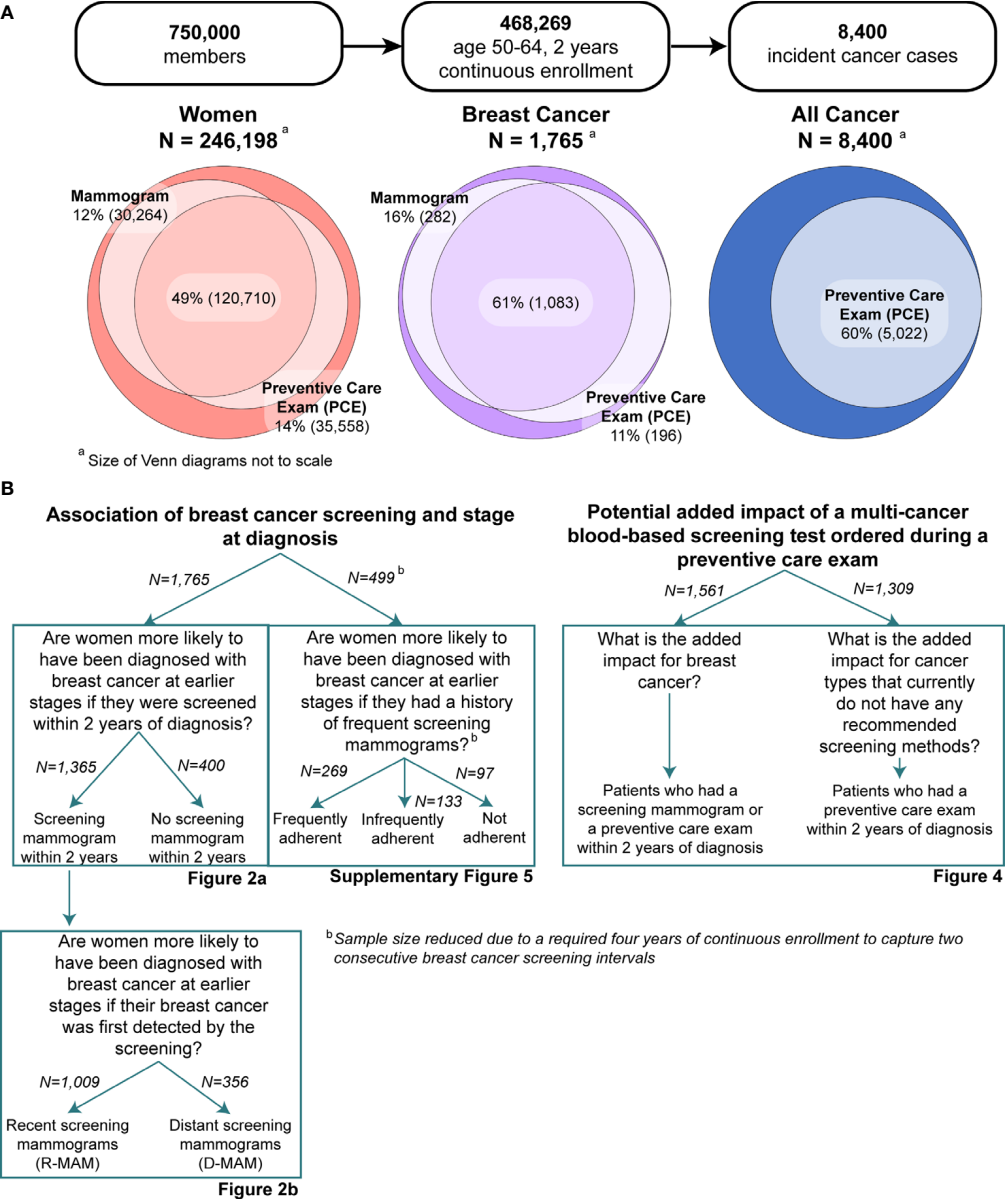
**(A)** Breakdown of the study into three subpopulations: women, all cancer, and breast cancer-specific populations. Inclusion criteria were ages 50-64 and at least 2 years of continuous enrollment. Screening mammogram and preventive care exam (PCE) utilization were characterized for all women and breast cancer populations, while only PCE utilization was analyzed for the all cancer (men and women) population. **(B)** Breakdown of the study into main aims and specific research questions, along with their associated figures. Note that analysis on the frequency of screening mammogram history required at least four years of continuous enrollment. A description of the methods for this analysis can be found in the **Supplementary Material**.

**Figure 1** illustrates the research questions and cohorts included in this study. In focusing specifically on breast cancer, we sought to test the association between presence and timing of screening mammography and breast cancer stage at diagnosis. We further distinguished between cancers associated with a recent screening mammogram (also referred to as R-MAM) versus those diagnoses occurring distant from the prior screening mammogram (also referred to as D-MAM). We then developed methods to characterize the potential for intervention *via* blood-based cancer screening by evaluating the frequency of PCEs preceding the diagnosis of breast cancer in women who did or did not undergo screening mammography. Using the same methods, we also described the proportion of individuals with any type of incident cancer who also had a PCE prior to their cancer diagnosis. This descriptive information may represent useful input for researchers and policymakers interested in understanding the real-world opportunity for adding MCED tests to the cancer screening paradigm for insured populations.

## Materials and Methods

### Data Sources

HIPAA-compliant, de-identified data were obtained from the BHI data set containing five years of claims (facility, professional, and pharmacy) for 750,000 enrolled primary members with medical and pharmacy benefits. The data set included only members with at least 24 continuous months of medical and pharmacy claims and did not include Medicare Advantage lives. The enrollment period spanned November 1, 2014 to October 31, 2019. Member information included sex, year of birth, rural-urban commuting area code (RUCA), and region. RUCA codes were aggregated into two categories for rural and urban areas using a published method (16).

Analyses were limited to eligible members aged 50-64 years at date of cancer diagnosis. 50 is the starting age at which USPSTF recommends screening mammograms, while 65 or older is the age of Medicare eligibility, a segment which is not part of this analysis (3).

### Identifying Incident Cancers

Previous research has suggested that claims data alone without linkage to a tumor registry may be insufficient to comprehensively characterize cancer incidence (17). However, other investigators have developed and validated algorithms to support reliable inference of cancer incidence and stage from claims data (7–11).

The incident cancer identification method used in this study is based on a series of definitions developed and validated by Setoguchi et al. (7) and replicated by Bronson et al. (8). While Setoguchi et al. proposed four possible definitions to identify incident cancer from claims data, their second approach (Definition 2; ≥2 cancer diagnosis codes within 2 months) was selected for this study due to its balance among sensitivity (78.89%), specificity (99.62%), and PPV (76.56%) (7). Additionally, this definition is intended to broadly identify all cancers rather than a single cancer type; Bronson et al. demonstrated that the performance of “Definition 2” remains consistent for breast, colorectal, and lung cancers (8).

All facility and professional claims were scanned for International Classification of Diseases, Ninth and Tenth Revision (ICD-9 and ICD-10) codes for malignant neoplasms, based on Surveillance, Epidemiology, and End Results Program (SEER) Casefinding Lists, excluding those of unspecified site, in the primary position of the claim (18). Inclusion criteria for incident cancer required at least 2 claims with a cancer ICD-9 or ICD-10 diagnosis code in the primary position, occurring on separate days and within two months of each other. The index or diagnosis date was defined as the date of the earliest claim that met the inclusion criteria. A retrospective claims review was used to confirm continuous enrollment and exclude misclassification of prevalent cancers, indicated by the presence of any claim with a diagnosis of cancer during a 12-month “look-back” period. A comprehensive list of ICD codes used in this study is available upon request.

### Incident Cancer Type

The incident cancer type was identified by the diagnosis code of the claim at the index date (96% of cancer patients). If there were multiple cancer types diagnosed at the index date, then the most frequently occurring cancer type diagnosed in all claims after the index date was identified as the incident cancer type (4% of cancer patients). If there were an equal number of claims for multiple cancer types, then each of the cancer types was considered a separate primary cancer (<0.01% of cancer patients).

### Staging Breast Cancer Cases

Incident breast cancers were staged using a claims-based algorithm developed by Blumen et al. to classify stage I/II, III, and IV breast cancers based on NCCN treatment guidelines (9). Per that method, stage IV breast cancer was classified by ≥2 claims at least 15 days apart for a secondary neoplasm in any position of the claim, occurring from 1 month before to 6 months after the index date. Cases not meeting the stage IV criteria were classified as stage III based on use of neoadjuvant therapy (≥2 claims for radiation and/or chemotherapy after the diagnosis date and before surgery) or axillary lymph node involvement (≥2 claims at least 15 days apart in any position of the claim and occurring within 6 months of the diagnosis date). Cases not meeting the stage IV or stage III criteria were classified with stage I/II disease. All codes used to identify breast procedures and diagnoses can be found in the **Supplementary Material**.

### Identifying Metastatic Cancers

While breast cancers were staged using an algorithm based on NCCN treatment guidelines, metastatic cancers were broadly identified for all cancer types based on ICD codes for secondary neoplasms (ICD-9 codes 197.xx-199.xx; ICD-10 codes C78.xx-C80.xx) or the presence of ICD codes for primary neoplasms at a different site from the original diagnosis (10). ICD codes indicating lymph node involvement were excluded as distinct secondary cancer sites since they are more indicative of regional rather than metastatic disease. These methods were generalized to all cancer types based on algorithms developed and validated by Nordstrom et al. (10).

### Comparisons With Nationally Representative Data

Cancer incidence in the BHI claims data determined using the published methods described above was compared with the incidence data from the SEER 18 database submitted in November 2018 and retrieved using SEER*Stat 8.3.8 software (19). Comparisons were made by age and cancer type.

Mammogram utilization in the BHI data, which was defined by the presence of a mammography claim within 24 months of the last date of enrollment, were compared with the results from the 2015 NHIS (20).

### Study Design and Populations

This study considered three subpopulations, depicted in **Figure 1A**: all women, women diagnosed with breast cancer, and all members with incident cancer. The first two subpopulations were analyzed for screening mammogram utilization and all three subpopulations were analyzed for PCE utilization within the two years prior to each member’s index date, defined as the date of diagnosis for incident cancer members or date of last enrollment for non-cancer members. Mammograms were identified by CPT/HCPCS codes for mammography and breast tomosynthesis, and were further subdivided into diagnostic and screening procedures. PCEs were identified by CPT/HCPCS codes for initial or established comprehensive preventive visits (**Supplementary Table 1**).

Inclusion in any of the three sub-populations required that members were aged 50-64 and enrolled for 2 continuous years in order to fully capture mammogram adherence, which is recommended by the USPSTF at two-year intervals, and PCE utilization (3). **Figure 1B** illustrates the two main aims of the study and the research questions that guided the analyses. The two aims were to understand the association of screening adherence and stage at diagnosis by focusing specifically on breast cancer, and to quantify the potential additive impact of a multi-cancer blood-based screening test ordered during a PCE. Screening adherence was characterized by the presence of a screening mammogram within a two-year window, a method that has also been used in previous breast cancer studies (21). However, other studies have also characterized frequency of screening by quantifying the time between two sequential screening mammograms (22). While not a primary research question in this study, we also sought to identify the association of screening mammogram frequency with stage at breast cancer diagnosis, and the methods are described in further detail in the **Supplementary Material**.

### Statistical Methods

The one-sample proportion test with Bonferroni correction was applied to test differences between BHI and SEER cancer incidence by cancer type and age. A Pearson chi-square test for independence was used to evaluate association of age, region, and RUCA category with mammogram utilization. Potential predictors of biennial mammogram utilization were investigated using multivariate logistic regression.

Within the breast cancer cohort, association between screening mammogram utilization and stage at breast cancer diagnosis was assessed using both the Pearson chi-square test for independence and univariate ordinal logistic regression. Age, region, RUCA category, and presence of a biennial screening mammogram were considered as potential predictors of stage at breast cancer diagnosis in the univariate ordinal logistic regression. In women who had a screening mammogram, time from the most recent mammogram to breast cancer diagnosis was modeled as a Gaussian mixture using the mixtools R package to explore the temporal pattern of mammography prior to cancer diagnosis (23). The cohort of patients with breast cancer and a screening mammogram were further divided into two sub-cohorts based on inference of two different underlying Gaussian distributions of time from screening to diagnosis. A Pearson chi-square test was used to evaluate association between these sub-cohorts and stage at breast cancer diagnosis. Univariate ordinal logistic regression was used to evaluate the odds of a later-stage tumor based on categorical and continuous representations of time from a screening mammogram to diagnosis. Polynomial regression was used to identify whether the categorical or continuous representation of time was a more significant predictor of stage at breast cancer diagnosis.

PCE utilization was characterized for all women during the two years before their index date. Multivariate logistic regression was used to evaluate association of age, region, RUCA category, and a screening mammogram with PCE utilization in all women aged 50-64. Comparable analyses were carried out in the all-cancer cohort. PCE utilization was also assessed specifically for patients identified with metastatic disease. Multivariate logistic regression was used to evaluate association of demographic factors with PCE utilization.

Statistical significance was indicated by a p <0.05. All analyses were performed using R version 3.6.3.

### Quantifying the Opportunity for an Additive Blood-Based Screening Test

In order to quantify the target opportunity of an additive blood-based screening test ordered during a PCE, we evaluated both current breast cancer screening rates and potential screening rates if there were widespread adoption of such a test. For breast cancer, the potential screening rate was the proportion of breast cancer patients who had either a screening mammogram or PCE in the two years before their diagnosis. For other cancer types, we looked at tumor types which make up the top 5 leading causes of cancer death without USPSTF-recommended screening modalities available. We limited this list to solid tumors, which are the focus of blood-based multi-cancer screening tests currently in development (12–14). Cancer types that meet these criteria include prostate, pancreatic, liver, lymphoma, and ovarian cancers (24). The potential screening rate for these cancers was defined as the proportion of patients with these tumor types that had a PCE in the two years before their diagnosis date. Colorectal, lung, and cervical cancers were not included in this analysis as their current screening rates were not studied with these data.

## Results

### Identifying Incident Cancer Cases

We identified 8,400 incident cancers from the BHI claims data, which included 1,765 breast cancer cases. 2016 SEER data was used as a nationally representative comparator. When stratified by age, the difference between BHI and SEER in overall cancer incidence was only nominally significant for patients age 55 (proportion test, adjusted p=0.04) (**Supplementary Figure 2**). When comparing incidence of individual cancer types, no significant difference was observed in the incidence per 100,000 between BHI and SEER for breast cancer (156 *vs.* 141, proportion test, adj. p=0.6). Of the cancer types that make up the top 5 leading causes of cancer death without a recommended screening modality, no significant frequency differences were observed for prostate, pancreatic, lymphoma, and ovarian cancers, while a significant difference was observed for liver cancer (adj. p <0.001) (**Supplementary Figure 3**).

### Mammogram Utilization

We assessed mammogram utilization in the BHI data and used the results from the 2015 NHIS as a nationally representative comparator (20). The NHIS does not separately report results for screening and diagnostic mammogram utilization, so for purposes of this comparison only, the BHI data were analyzed for both screening and diagnostic mammograms. **Table 1** shows overall combined mammogram utilization by age, region, and RUCA category, compared with the 2015 NHIS. Mammogram utilization was significantly associated with age, region, and RUCA category (Pearson chi-square test, p <0.001 for all demographic factors). Multivariate logistic regression showed that when compared to a reference group aged 50-54 and located in the urban Northeast, the odds of undergoing mammography was greater for women aged 55-59 and 60-64, lower for women located in the Midwest, West, and South, and lower for those located in rural areas (multivariate logistic regression, p <0.05 for all demographic factors) (**Supplementary Table 2**). Overall mammogram utilization for women aged 50-64 in the BHI data was lower than the 2015 NHIS data, even when including diagnostic mammograms (62% *vs.* 70%, **Table 1**, Pearson chi-square test, p <0.001).

**Table 1:** Screening and diagnostic mammogram utilization by age, region, and RUCA category.

	N	Total	Percent (95% CI)	P-value^b^
Age group				<0.001
50-54 years	46,859	77,653	60.3 (60.0-60.7)	
55-59 years	53,043	85,562	62.0 (61.7-62.3)	
60-64 years	53,221	82,983	64.1 (63.8-64.5)	
Region				<0.001
Northeast	36,779	56,950	64.6 (64.2-65.0)	
Midwest	37,405	60,624	61.7 (61.3-62.1)	
West	14,837	24,802	59.8 (59.2-60.4)	
South	64,033	103,667	61.8 (61.5-62.1)	
Other	69	155	44.5 (36.7-52.3)	
RUCA category				<0.001
Urban	132,341	212,215	62.4 (62.2-62.6)	
Rural	20,502	33,515	61.2 (60.7-61.7)	
Other	280	468	59.8 (55.4-64.3)	
Overall utilization				<0.001
BHI	153,123	246,198	62.2 (62.0-62.4)	
Screening	150,974	246,198	61.3 (61.1-61.5)	
Diagnostic	5,572	246,198	2.3 (2.2-2.3)	
2015 NHIS^a^	3,036	4,314	70.4 (69.0-71.7)	

CI, confidence interval; NHIS, National Health Interview Survey; RUCA, Rural-Urban Commuting Area Code.

a Data Source: NCHS, National Health Interview Survey, 2015.

b P-value from Pearson chi-square test for independence.

### Association of Screening Mammogram Utilization and Stage at Breast Cancer Diagnosis

**Figure 2:**
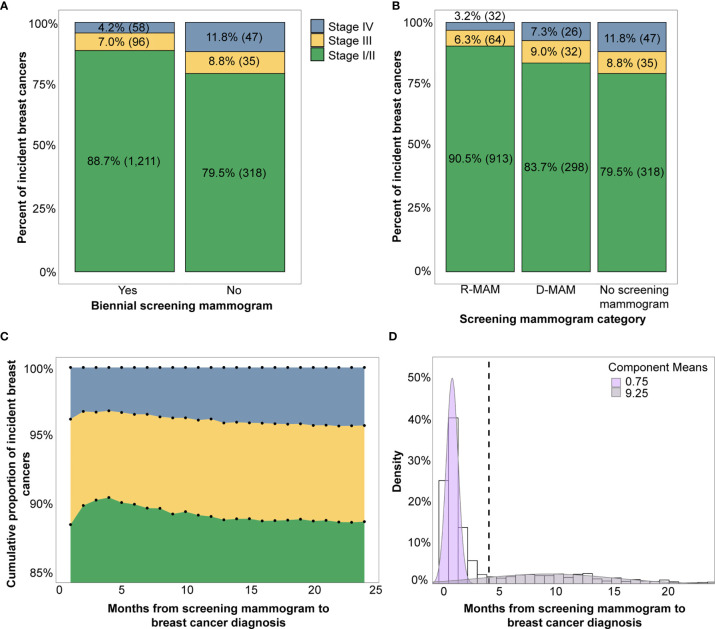
**(A)** Breast cancer stage distribution by presence of a biennial screening mammogram (yes *vs.* no) and **(B)** Breast cancer stage distribution by time from screening mammogram grouped into R-MAM (screening mammogram <4 months from diagnosis) and D-MAM (screening mammogram 4-24 months from cancer diagnosis). **(C)** The cumulative proportion of each stage of incident breast cancers by months from the screening mammogram to breast cancer diagnosis. **(D)** Distribution of months from screening mammogram to breast cancer diagnosis is bimodally distributed and modeled by a two-component Gaussian mixture. Note that percentages may not add to 100% due to rounding.

To assess the association between breast cancer screening behavior and breast cancer stage at diagnosis, women with incident breast cancer were evaluated for evidence of a screening mammogram claim in the two years before diagnosis. Women with screening mammograms had a significantly lower proportion of advanced stage diagnoses (stage III or IV) compared to those who did not have a screening mammogram (11% *vs.* 21%, Pearson chi-square test, p <0.001) and the odds of a later-stage breast cancer diagnosis were 2.10 fold greater for women without a screening mammogram (univariate ordinal logistic regression, p <0.001) (**Figure 2A**, **Table 2**).

**Table 2:** Univariate ordinal logistic regression analysis on the odds of having a later stage breast cancer diagnosis for women aged 50-64 using age group, region, RUCA category, and (analysis 1) presence of a screening mammogram or (analysis 2) grouped and continuous time from screening to diagnosis as predictors.

	Analysis 1: Biennial screening mammogram				Analysis 2: Time from screening to diagnosis		
	OR	95% CI	P-value		OR	95% CI	P-value
Age group (ref. 50-54 years)							
55-59 years	0.98	(0.70-1.38)	0.91		0.98	(0.70-1.38)	0.91
60-64 years	0.82	(0.59-1.15)	0.26		0.82	(0.59-1.15)	0.26
Region (ref. Northeast)							
Midwest	1.46	(0.93-2.12)	0.07		1.46	(0.93-2.12)	0.07
West	1.38	(0.76-2.33)	0.25		1.38	(0.76-2.33)	0.25
South	1.16	(0.80-1.72)	0.28		1.16	(0.80-1.72)	0.28
RUCA (ref. Urban)							
Rural	1.24	(0.84-1.78)	0.26		1.24	(0.84-1.78)	0.26
Screening mammogram (ref. Yes)							
No	**2.10**	**(1.56-2.81)**	**<0.001**				
Time between screening mammogram and breast cancer diagnosis (ref. R-MAM)							
D-MAM					**1.87**	**(1.31-2.65)**	**<0.001**
No screening mammogram					**2.55**	**(1.85-3.51)**	**<0.001**
Time between screening mammogram and breast cancer diagnosis (ref. D-MAM)							
R-MAM					**0.53**	**(0.38-0.76)**	**<0.001**
No screening mammogram					1.36	(0.94-1.98)	0.10
Time between screening mammogram and breast cancer diagnosis (continuous)					**1.03**	**(1.00-1.06)**	**0.03**

ref, reference; OR, odds ratio; CI, confidence interval; R-MAM, screening mammogram <4 months from diagnosis; D-MAM, screening mammogram 4-24 months from diagnosis.

Boldface indicates p < 0.05.

Of the women with breast cancer who had received a screening mammogram, the distribution of time from screening mammogram to breast cancer diagnosis was approximated using a two-component Gaussian mixture model (**Figure 2D**). The bimodal distribution had its first component centered at 0.75 months and the second component centered at 9.25 months, suggesting two patterns of routine mammograms prior to cancer diagnosis. The cumulative proportion of stage I/II breast cancers increased between 0 to 3 months, reached a peak at 4 months, and then gradually declined (**Figure 2C**). Comparatively, stage III and IV breast cancers decreased to a minimum at 4 months with a subsequent gradual increase. Based on these observations, we selected 4 months as the cutoff point at which to separate the two components of the Gaussian mixture model.

When we further sub-divided the USPSTF guideline adherent women into two groups – those with a recent screening mammogram <4 months prior to diagnosis (R-MAM) and those with a distant screening mammogram 4-24 months prior to diagnosis (D-MAM), there was a significant difference in breast cancer stage at diagnosis (Pearson chi-square test, p <0.001). Women with R-MAMs had the highest proportion of stage I/II breast cancers (91%) (**Figure 2B**). Women with D-MAMs were associated with a 87% increased odds of a later-stage (III or IV) breast cancer diagnosis as compared to women with R-MAMs (univariate ordinal logistic regression, p <0.001, **Table 2**), while no screening mammogram in the previous 24 months was associated with a 155% increased odds of a later-stage breast cancer diagnosis compared with R-MAMs (univariate ordinal logistic regression, p <0.001, **Table 2**). Notably, the increased odds of a later-stage diagnosis when the no screening mammogram group was compared with the D-MAMs was not significant, with an odds ratio of 1.36 and two-sided 95% confidence interval of (0.94, 1.98) (univariate ordinal logistic regression, p=0.10, **Table 2**). It was also observed that women in the R-MAM group had a subsequent diagnostic mammogram an average of 0.5 months following the screening, whereas the average time to a diagnostic mammogram was 10.2 months for women in the D-MAM group (**Supplementary Figure 4**).

When time between screening mammogram and breast cancer diagnosis was used as a continuous predictor, a one-month increase was associated with a 3% increase in the odds of an advanced stage diagnosis (univariate ordinal logistic regression, p=0.03, **Table 2**). A polynomial regression model was also fitted, including a 2^nd^ degree term of time from screening mammogram to breast cancer diagnosis. The coefficient for the 2^nd^ degree term was not significant (p=0.33). The nominal significance of the regression analysis suggests that the grouped time from screening mammogram to diagnosis is a more significant predictor of stage at breast cancer diagnosis. Age group, region, and RUCA category did not significantly impact breast cancer stage at diagnosis (**Table 2**).

Analysis was also performed to identify the association of screening mammogram frequency with stage at breast cancer diagnosis (methods in **Supplementary Material**). While a Pearson chi-square test identified a significant association (p=0.005, **Supplementary Figure 5**), univariate ordinal logistic regression analysis was not significant (**Supplementary Table 3**). It showed that being infrequently adherent to breast cancer screening was associated with a 31% increased odds (p=0.4) and no screening history was associated with an 82% increased odds (p=0.08) of a later stage diagnosis as compared with women frequently adherent to breast cancer screening. Notably, the size of the cohort in this analysis was considerably reduced from 1,765 women to 499 women due to the requirement of four years of continuous enrollment prior to diagnosis, which was necessary to capture two consecutive screening events.

### Preventive Care Exam Characterization and the Opportunity for an Additive Blood-Based Screening Test

**Figure 3:**
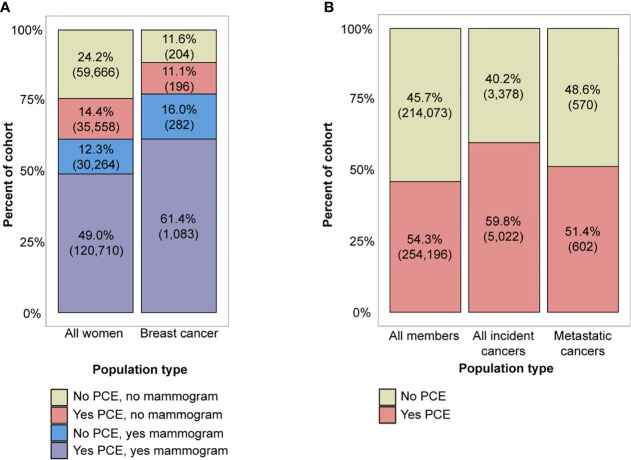
**(A)** Combinations of preventive care exam (PCE) and screening mammogram utilization in the all women aged 50-64 and breast cancer cohorts, and **(B)** PCE utilization in the all member aged 50-64, incident cancer, and metastatic cancer cohorts. Note that percentages may not add to 100% due to rounding.

While a large fraction of all women (61%, N=150,974) and women with breast cancer diagnoses (77%, N=1,365) had a screening mammogram within 2 years of their index date, which was the last date of enrollment for women without cancer or date of diagnosis for women with cancer, a similar number (63% of all women and 73% of incident breast cancer patients) had a PCE (**Figure 3A**). Multivariate logistic regression analysis showed that age, region, RUCA category, and a biennial screening mammogram claim were significantly associated with having at least one PCE in two years (**Supplementary Table 4**), with women having the highest rates of PCEs in younger age groups, the Northeast, urban areas, and when there was also a screening mammogram.

Finally, we looked at the full incident cancer subpopulation relative to PCE utilization in the two years prior to cancer diagnosis to assess the opportunity for a multi-cancer screening test for all cancer types, including breast cancer. The majority of all members aged 50-64 (54%, N=254,196), incident cancer patients (60%, N=5,022), and metastatic cancer patients (51%, N=602) had a history of at least one PCE in the two years prior to diagnosis (**Figure 3B**). PCE utilization was lower in patients with a metastatic stage (51%) compared to patients with a non-metastatic stage (61%) (Pearson chi-square test, p <0.001). A breakdown of PCE utilization by cancer type can be found in **Supplementary Figure 6**. Multivariate logistic regression analysis showed that in the cohort of all cancer patients, age, region, and RUCA category were significantly associated with having a PCE (**Supplementary Table 5**). When compared to a reference group aged 50-54 and located in the urban Northeast, greater age, rural areas, and locations in the South, West, and Midwest were associated with lower odds of having had a PCE (multivariate logistic regression, p <0.001 for all demographic factors excluding Midwest locations).

**Figure 4:**
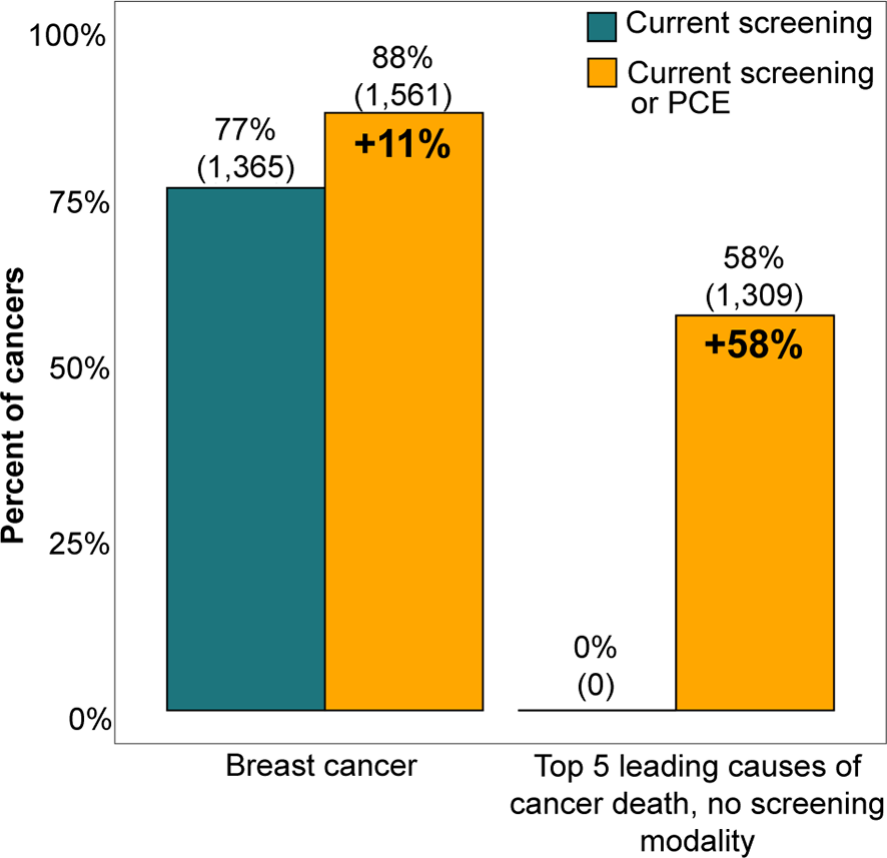
Current screening rates for breast cancer and the top 5 cancers that are both the leading causes of cancer death and lack a USPSTF recommended screening modality were compared with the potential screening rates possible with an additive multi-cancer screening blood test performed at a preventive care exam (PCE). The potential screening rate for breast cancer was the proportion of breast cancer patients who had either a screening mammogram or PCE in the two years before their diagnosis date, while the potential screening rate for other cancer types was the proportion of patients who had a PCE in the two years preceding diagnosis. The top 5 cancers that are leading causes of cancer death were limited to cancer types that do not currently have a screening modality and that form solid tumors, which include prostate, pancreatic, liver, lymphoma, and ovarian cancers (24). Conclusions could not be made for colorectal, lung, and cervical cancers because their screening adherence rates were not explored in this study.

The relationship between PCEs and cancer diagnosis represents an opportunity for additional multi-cancer screening with a blood-based test. **Figure 4** illustrates that while 77% of breast cancer cases had a biennial screening mammogram, a multi-cancer screening assay ordered during a PCE could have screened up to an additional 11% of breast cancer cases and up to 58% of the top 5 cancers that are leading causes of cancer death currently without a USPSTF-recommended screening modality, assuming full compliance. These represent patients who had a history of at least one PCE in the two years before diagnosis.

## Discussion

Using a private payer claims database, we demonstrated that women who underwent a screening mammogram and were subsequently diagnosed with breast cancer in the ensuing four months had earlier stage diagnoses (stages I and II) than did women who either did not have any mammograms in the 24 months prior to their diagnosis or who received mammograms in months 4-24 prior. Women in these two latter groups were frequently diagnosed with more advanced stages of breast cancer (stages III and IV), but the majority (65%) were observed to have had a PCE in the 24 months prior to their diagnosis, illustrating the potential for delivery of additional cancer screening tests that could be ordered in the context of a routine preventive care visit.

Significant associations were observed between mammography utilization and age group, region, and RUCA category; these results are consistent with Khushalani et al., who demonstrated that age, region, and metropolitan status are associated with mammography screening utilization (22). However, overall BHI mammogram utilization was lower than was observed in the 2015 NHIS. This difference may be attributable to overestimation in NHIS, as suggested by previous studies comparing claims with survey estimates. Specifically, Randolph et al. noted that screening mammogram utilization rates based on claims are generally lower than estimates from national surveys due to errors in self reports and inaccurate recall of previous mammograms (25).

The association of screening mammogram utilization with earlier stage at breast cancer diagnosis was significant. Within the incident breast cancer population, those without a screening mammogram had increased odds of a later-stage diagnosis. This is consistent with the USPSTF evidence review of breast cancer screening which identified a reduced risk of advanced cancer (stage III and IV) for women age 50 and older receiving biennial screening mammograms (3). Reviewers also noted that screened women were more likely to have their tumors surgically resected and have breast conserving surgery, a finding that supports the use of screening interventions to reduce morbidity from breast cancer (3).

The elapsed time between screening mammogram and date of diagnosis was also associated with cancer stage. Based on the bimodal distribution observed in the time from screening mammogram to diagnosis, a 4-month cutoff was applied that subdivided the women who had a biennial screening mammogram into R-MAMs and D-MAMs, which is also supported by previous studies. Fenton et al. identified 123 days (4.1 months) as the point that separates screening-detected *vs.* non-screening-detected breast cancer, a value that was validated and generated an optimal classification of cases in their algorithm (26). The association of R-MAMs and D-MAMs with stage at diagnosis is also consistent with Niraula et al., who found that women with interval breast cancers were diagnosed with higher-grade tumors and higher hazards of death compared to those with screen-detected breast cancers (21).

Consistent with these previous studies, we hypothesize that the R-MAMs represent screen-detected breast cancers and that their screening mammograms resulted in clinically appropriate follow-up and an eventual breast cancer diagnosis. Comparatively, D-MAMs represent interval breast cancers and that their screening mammograms likely did not lead to an immediate diagnostic follow-up (e.g., the mammogram did not show suspicious results at the time and the breast cancer was instead diagnosed after the onset of symptoms or the mammogram was a true-positive but there was an unexpected delay in cancer diagnosis). These hypotheses are supported by observations of diagnostic mammograms occurring shortly after a screening mammogram in the R-MAM group and over a longer period of time in the D-MAM group (**Supplementary Figure 4**), further analysis would be needed linking claims data to electronic health records to conclusively confirm these hypotheses.

With the emergence of genomics technologies that enable multi-cancer screening, there exists an opportunity to introduce additional cancer screening to more individuals through an additive blood-based test ordered during PCEs, exams which typically involve other blood tests for biomarkers like HbA1c and serum cholesterol. With respect to breast cancer, an additional blood-based screening test has the opportunity to incrementally benefit women who underwent both a screening mammogram and PCE (61% of women with breast cancer), while the potential benefit is enhanced for those who did not have a screening mammogram but did have a PCE (11% of women with breast cancer). Overall, 73% of women with breast cancer had the potential to benefit from a MCED test.

Beyond breast cancer, a multi-cancer blood-based screening test holds potential for much wider benefit, especially for patients diagnosed with cancer types that do not currently have USPSTF-recommended screening modalities available. Of all the women who were diagnosed with a cancer without a recommended screening modality (45% of female cancer patients), 55% had both a screening mammogram and PCE, while 13% had only a routine PCE in the two years before diagnosis. This highlights the fact that a majority of these women were, in fact, adherent to USPSTF recommendations for breast cancer and received preventive care but could not be screened for the cancer they later developed as there was no screening modality available. Expanding to both sexes and all cancers, 64% of cancer patients observed in the BHI claims data were diagnosed with a cancer that did not have a USPSTF-recommended screening test, yet 58% of these patients had a PCE.

Similar to the significant association between mammography and breast cancer stage evaluated in this study, we hypothesize that a highly specific multi-cancer blood-based screening test, when incorporated into routine care, has the potential to reduce the probability of other cancers being diagnosed at a more advanced stage. This is significant because studies have shown that cancers detected at earlier stages have better prognoses, improved survival, and lower associated costs (2, 27–30). Unfortunately, cancer types such as ovarian and pancreatic cancer, which do not have screening modalities, are typically diagnosed at advanced stages due to late symptom presentation (30). A distant stage ovarian cancer diagnosis has a 54% 5-year-survival rate, but this is increased to 98% with a localized diagnosis (2). Likewise, distant stage pancreatic cancer 5-year survival is 3%, but it is 39% for localized stages, further highlighting the unmet need and opportunity in cancer screening (2).

The opportunity to improve the scope and uptake of cancer screening tests is evident (12–14). However, a multi-cancer screening blood test will have to demonstrate high specificity to minimize the potential for false-positive results and the associated risks and costs of putting patients through unnecessary diagnostic procedures. Large randomized trials will be required to determine test performance characteristics in presumably healthy individuals, as well as the benefits, harms and costs of introducing a novel cancer screening test to patients and the healthcare system. Preliminary studies have been conducted supporting these findings and further trials are ongoing or in the final planning stages, so the results of this study are timely for decision-makers interested in planning for market introduction of these novel tests (13). As this study highlights, there is an added opportunity to target PCEs, or even enable access through local, community, or at-home blood draw services, but this will require large efforts into investigating how this can be integrated into routine care. This includes patient and provider education and shared decision-making tools, seamless workflows to minimize burden on primary care providers, adequate explanation of results and implications, as well as guidance and care coordination support to ensure effective follow-up and continued engagement in cancer screening and preventive health.

There are several limitations to this study. A key limitation is that we did not have the ability to link insurance claims with a tumor registry, meaning that cancer identification and staging were inferred based on ICD diagnosis codes. The algorithm used for cancer identification has a high specificity but a lower sensitivity and PPV, suggesting that this study is under-counting the number of incident cancers (7). Additionally, Chawla et al. suggested that the use of claims data alone to infer cancer stage at diagnosis misclassifies a significant number of patients (17). They note that algorithms that use claims data to stage cancers have shown to overestimate the frequency of localized cancers and underestimate regional cancers when compared with SEER data (17). This likely suggests that we not only underestimated the number of cancers, but we also underestimated the proportion of stage III and IV breast cancers, which could affect the association between screening and stage at diagnosis.

Second, a limitation inherent in claims data is that it is not possible to identify the outcome of screening or diagnostic procedures. Results of tests or imaging are not included in medical claims, so we were unable to conclusively identify screening-detected breast cancers. While inferences were made based on the timing of diagnostic mammograms following screening, studies using EMR data in conjunction with claims data are more likely to enable conclusions about specific procedures leading up to diagnosis. Additionally, genetic information or family history data was not available in this data set, so they could not be included as possible confounders for stage at breast cancer diagnosis, or as factors associated with screening mammogram or PCE utilization. 

Third, because the data set was limited to a five-year time window, the study was unable to fully address the association of breast cancer screening frequency with stage at diagnosis due to a small sample size of breast cancer patients with four years of continuous enrollment preceding diagnosis. Future studies with access to an expanded data set would enable statistical power for this analysis.

Finally, this study analyzed commercial claims data, which are not representative of a large proportion of the population that could stand to benefit from a blood-based screening test. Future studies using Medicare claims data will prove valuable in understanding cancer screening potential in the population age 65 and older. Additionally, the data analyzed in this study contained limited member demographic factors. Future studies should consider the impact of all factors influencing health (social, economic, behavioral, etc.) on patterns of screening utilization, and additional opportunities for intervention beyond routine PCEs that ensure equitable access and follow-up support.

Despite these limitations, we demonstrated that it is possible to use a longitudinal claims data set to determine the association between mammography screening and stage of breast cancer at diagnosis. Moreover, we illustrated the potential for use of PCEs to afford timely opportunities to detect additional cancers, ideally at earlier stages when treatments are less expensive and potentially curative. This information should prove useful to researchers and policymakers interested in assessing the clinical utility (net benefits) of novel screening tests for earlier cancer detection.

## Data Availability Statement

Access to claims data was awarded to Thrive Earlier Detection by Blue Health Intelligence (BHI) as terms of the BlueCross BlueShield Data Innovation Challenge. Blue Health Intelligence is an independent licensee of Blue Cross Blue Shield Association (BCBSA). BCBSA is an association of independent, locally operated Blue Cross and Blue Shield companies. Thrive competed in and won the Challenge in 2019. Requests to access these datasets should be directed to info@bluehealthintelligence.com.

## Author Contributions

AC and AW participated in the BlueCross BlueShield Data Innovation Challenge, resulting in the acquisition of the BHI claims data. CH and AC conceptualized and designed the study. PP provided support and feedback in the analysis of the claims data. CH and AC analyzed and interpreted the data. JW provided statistical analysis support. CH wrote the first draft of the manuscript. PP, YL, JW, SA, AP, AW, PD, and AC provided critical feedback and support throughout the data analysis and manuscript revision process. All authors contributed to the article and approved the submitted version.

## Funding

This study was supported by Thrive, An Exact Sciences Company. The funder was not involved in the study design, collection, analysis, interpretation of data, the writing of this article or the decision to submit it for publication.

## Conflict of Interest

CH, YL, JW, SA, AP, AW, and AC were employed by Thrive, An Exact Sciences Company and hold equity and/or stock options in Exact Sciences. PP was employed by Blue Health Intelligence. PD was employed by the company Deverka Consulting.
