# Supplementary material for: Association of Breast Cancer Screening Behaviors With Stage at Breast Cancer Diagnosis and Potential for Additive Multi-Cancer Detection via Liquid Biopsy Screening: A Claims-Based Study
Source: Front Oncol. 2021 Jun 15;11:688455. doi: 10.3389/fonc.2021.688455 (PMC8241562; doi:10.3389/fonc.2021.688455)
Supplement: Supplementary file 1 [file DataSheet_1.pdf]

Supplementary Material

Supplementary Methods

Association of Screening Frequency and Stage at Breast Cancer Diagnosis

In this study, the presence of a screening mammogram claim was identified for breast cancer patients in a two-year window before diagnosis. The presence of a screening mammogram as well as the time from screening to diagnosis were found to be predictors for stage at breast cancer diagnosis. However, there are other methods of characterizing adherence to breast cancer screening recommendations. Previous studies have also characterized screening mammogram adherence by the frequency of screening mammogram utilization (1). In other words, characterizing whether patients were adherent to two subsequent mammograms. We also sought to identify the association of screening mammogram frequency with stage at breast cancer diagnosis.

The method of this additional analysis differs from the main analysis in that it requires that members have at least four years of continuous enrollment, rather than two years, in order to capture two complete screening intervals. This results in a reduction of the breast cancer cohort from 1,765 women in the original analysis to 499 women. Frequency for each patient was characterized as follows:

- Frequently adherent: screening mammogram within two years of diagnosis and also adherent to the previous screening mammogram.
- Infrequently adherent: screening mammogram within two years of diagnosis and not adherent to the previous screening mammogram, or screening mammogram not within two years of diagnosis and there is a record of a previous mammogram within two years.
- Not adherent: no history of a screening mammogram, or does have a record of a screening mammogram but it is not within two years of diagnosis and there is no record of a previous mammogram within two years.

This method of characterizing the frequency of screening mammogram adherence is similar to the method used by Khushalani et al (1). 269 women were identified as frequently adherent, 133 women as infrequently adherent, and 97 women as not adherent to breast cancer screening.

Supplementary Tables

Supplementary Table 1. CPT, HCPCS, ICD-9, and ICD-10 diagnosis codes used to identify procedures for breast cancer staging and routine care characterization.

Description	Codes
Breast cancer metastasis	**ICD-9 diagnosis codes:** 196.0x-196.2x, 196.5x-196.9, 197.xx, 198.xx<br>**ICD-10 diagnosis codes:** C77.0-C77.2, C77.4-C77.9, C78.xx, C79.xx, C7B.xx
Breast cancer surgery	**CPT/HCPCS codes:** 19120, 19125, 19126, 19160, 19162, 19180, 19182, 19200, 19220, 19240, 19297, 19301, 19302, 19303, 19304, 19305, 19306, 19307
Chemotherapy	Oral chemotherapy NDCs were obtained from SEER*Rx Antineoplastic Drugs Database (2).<br>**CPT/HCPCS codes:** J9000-J9999, J8510, J8520, J8521, J8530, J8560, J8561, J8565, J8600, J8610, J8700, J8705, J8999
Radiation therapy	**CPT/HCPCS codes:** 76370, 76950, 76965, 77014, 77261-77263, 77280-77299, 77300-77370, 77371-77399, 77401-77417, 77418, 77421, 77422-77423, 77427-77499, 77520-77525, 77600-77620, 77750-77799, 79005, 0197T, 0394T, 0395T, A9517, C1715-C1719, C1728, C2616, G0339-G0340, G6001-G6017
Axillary lymph node involvement	**ICD-9 diagnosis codes:** 196.3<br>**ICD-10 diagnosis codes:** C77.3
Diagnostic mammogram	**CPT/HCPCS codes:** 77051, 77055, 77056, 77061, 77062, 77065, 77066, G0204, G0206, G0279
Screening mammogram	**CPT/HCPCS codes:** 77052, 77057, 77063, 77067, G0202
Preventive care exam (PCE)	**CPT/HCPCS codes:** 99386, 99387, 99396, 99397

Supplementary Table 2. Multivariate logistic regression analysis on the odds of having undergone a screening or diagnostic mammogram within two years for women age 50-64 using age group, region, and RUCA category as predictors.

	**OR**	**95% CI**	**P-value**
Age group (ref. 50-54 years)			
55-59 years	1.07	(1.05-1.09)	<0.001
60-64 years	1.16	(1.14-1.18)	<0.001
Region (ref. Northeast)			
Midwest	0.89	(0.87-0.91)	<0.001
West	0.83	(0.80-0.85)	<0.001
South	0.88	(0.86-0.90)	<0.001
RUCA (ref. Urban)			
Rural	0.97	(0.95-0.99)	0.01

ref, reference; OR, odds ratio; CI, confidence interval.

Supplementary Table 3. Univariate ordinal logistic regression analysis on the odds of having a later stage breast cancer diagnosis for women age 50-64 using age group, region, RUCA category, and frequency of mammogram adherence as predictors.

	OR	95% CI	P-value
Age group (ref. 50-54 years)			
55-59 years	1.15	(0.61-2.18)	0.67
60-64 years	0.66	(0.33-1.32)	0.24
Region (ref. Northeast)			
Midwest	1.11	(0.53-2.38)	0.78
West	0.82	(0.25-2.29)	0.71
South	0.87	(0.44-1.79)	0.70
RUCA (ref. Urban)			
Rural	1.24	(0.59-2.41)	0.55
Screening mammogram frequency (ref. Frequently adherent)			
Infrequently adherent	1.31	(0.69-2.44)	0.40
Not adherent	1.82	(0.92-3.49)	0.08

ref, reference; OR, odds ratio; CI, confidence interval.

Supplementary Table 4. Multivariate logistic regression analysis on the odds of having undergone a preventive care exam (PCE) within two years for women aged 50-64 using age group, region, RUCA category, and biennial screening mammogram as predictors.

	**OR**	**95% CI**	**P-value**
Age group (ref. 50-54 years)			
55-59 years	0.88	(0.86-0.90)	<0.001
60-64 years	0.82	(0.80-0.84)	<0.001
Region (ref. Northeast)			
Midwest	0.93	(0.90-0.95)	<0.001
West	0.63	(0.61-0.65)	<0.001
South	0.92	(0.90-0.94)	<0.001
RUCA (ref. Urban)			
Rural	0.67	(0.65-0.68)	<0.001
Screening mammogram (ref. Yes)			
No	0.15	(0.14-0.15)	<0.001

ref, reference; OR, odds ratio; CI, confidence interval.

Supplementary Table 5. Multivariate logistic regression analysis on the odds of having undergone a preventive care exam (PCE) within two years for all members with an incident cancer diagnosed at 50-64 years using age group, region, and RUCA category as predictors.

	OR	95% CI	P-value
Age group (ref. 50-54 years)			
55-59 years	0.83	(0.74-0.94)	0.003
60-64 years	0.81	(0.72-0.91)	<0.001
Region (ref. Northeast)			
Midwest	0.95	(0.83-1.08)	0.4
West	0.64	(0.54-0.77)	<0.001
South	0.75	(0.67-0.84)	<0.001
RUCA (ref. Urban)			
Rural	0.68	(0.60-0.77)	<0.001

ref, reference; OR, odds ratio; CI, confidence interval.

Supplementary Figures

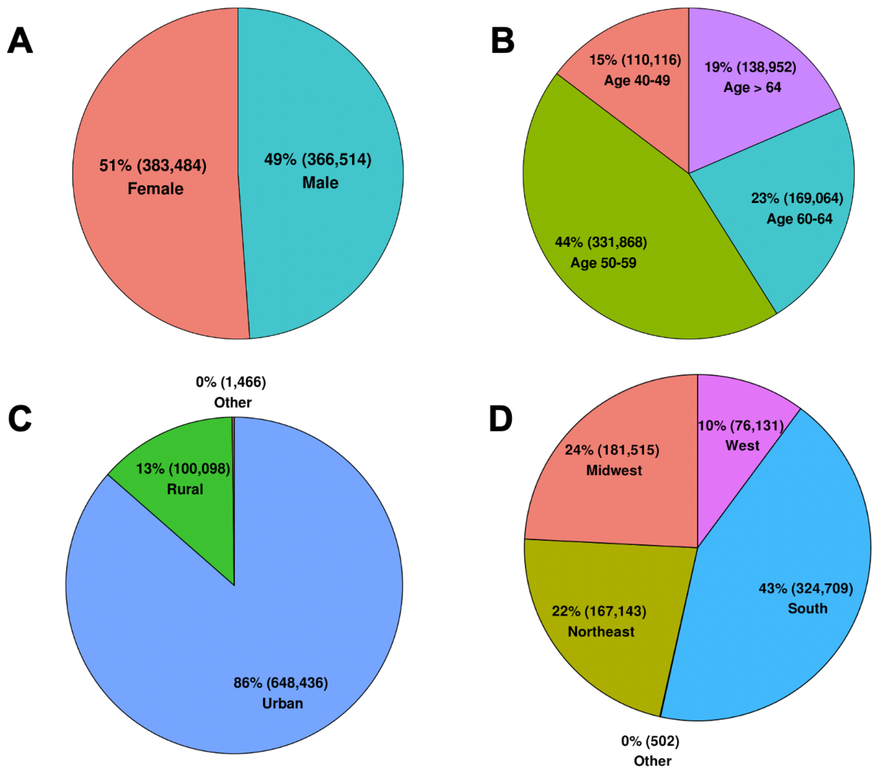

Supplementary Figure 1. Distributions for (A) sex, (B) age group, (C) RUCA category, and (D) region in the BHI data.

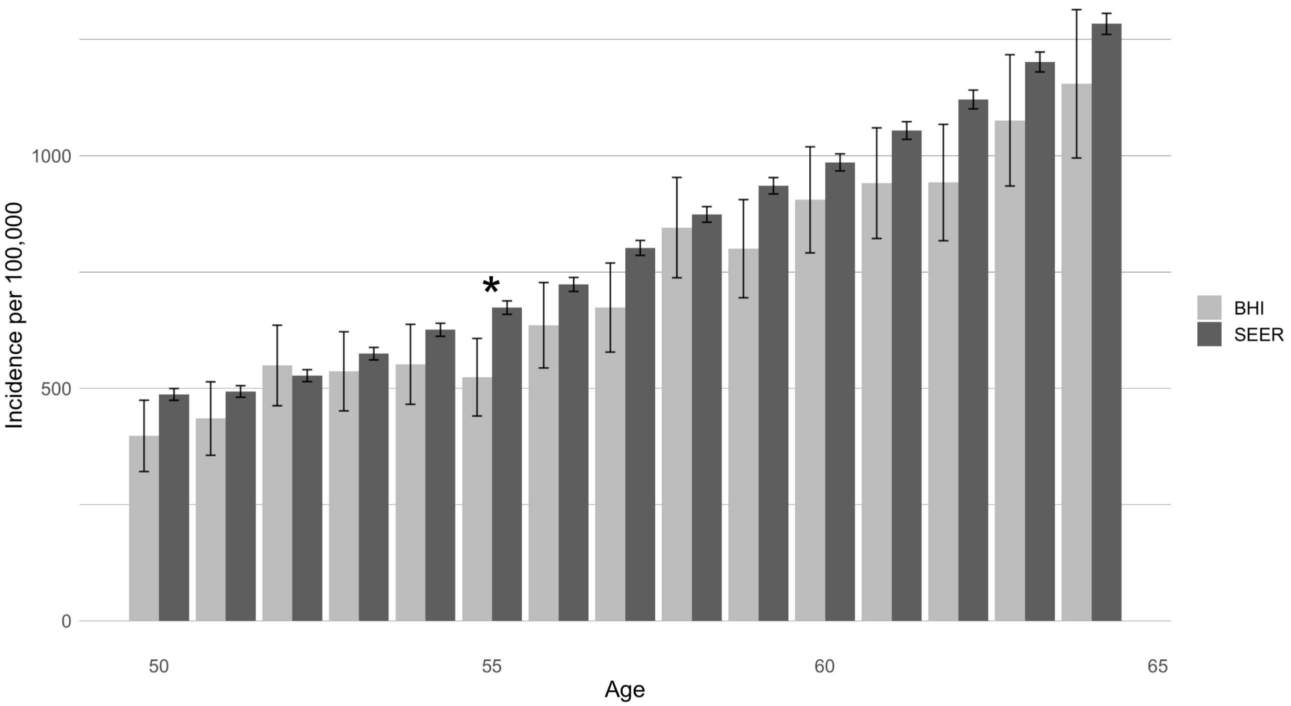

Supplementary Figure 2. BHI and SEER incidence per 100,000 in 2016 by age with two-sided 95% confidence intervals (CI). A two-sample proportion test with Bonferroni correction was applied to test the difference between BHI and SEER incidence. Significance (adjusted p <0.05) is noted with an asterisk.

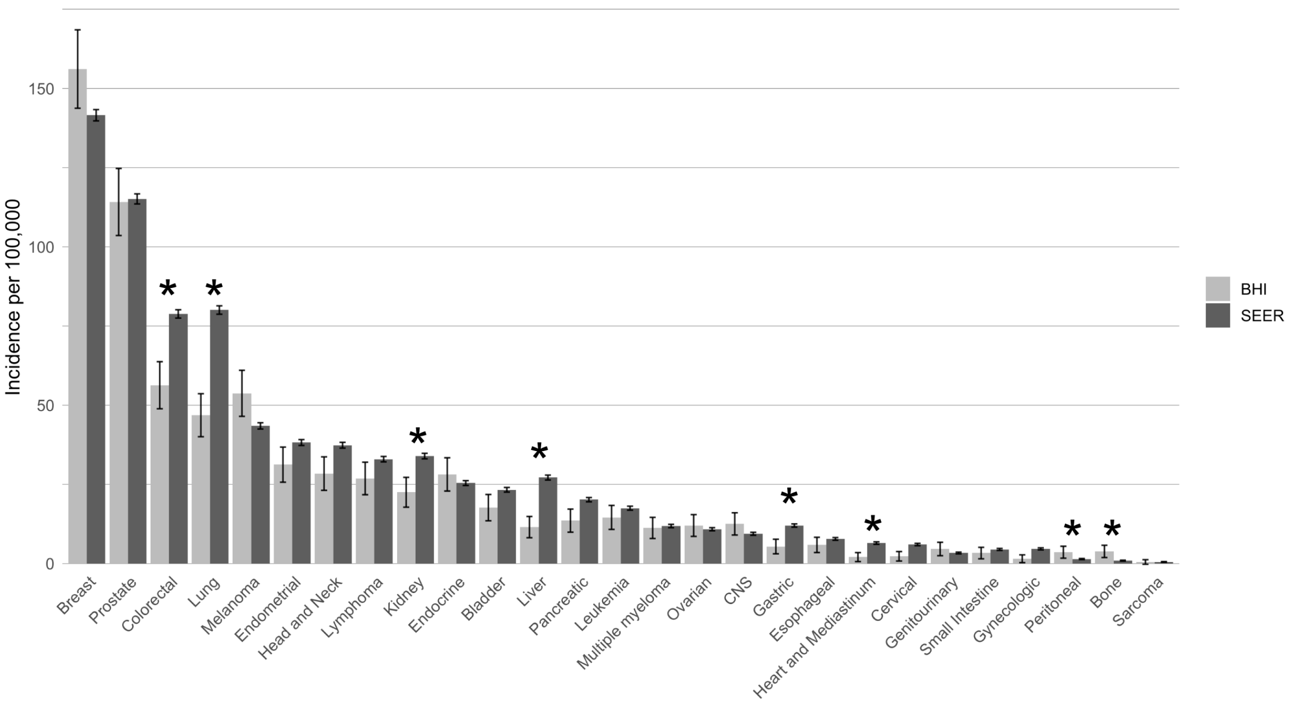

Supplementary Figure 3. BHI and SEER incidence per 100,000 for each cancer type in individuals aged 50-64 in 2016 with two-sided 95% confidence intervals (CI). The proportion test with Bonferroni correction was applied to test the difference between BHI and SEER incidence. Significance (adjusted $p < 0.05$) is noted with an asterisk. We noted significant differences between BHI and SEER incidence data for several prevalent (colorectal, lung, kidney, liver, gastric) and rare (heart and mediastinum, peritoneal, and bone) cancer types. One potential reason for the lower incidence in BHI members is that their claims data represent a privately insured population, while SEER data also include Medicaid and non-insured populations. One prior study by Grant et al. specifically investigated the distribution of incident cancer types by insurance status and found that incident lung, stomach, and liver cancers were among the five most common diagnoses in Medicaid recipients and uninsured persons (3). Differences between BHI and SEER incidence may also be attributed to the generalized method of incident cancer identification from the claims data; there may be nuances in how procedures are billed for different cancer types that the generalized method used in this study for cancers overall does not capture.

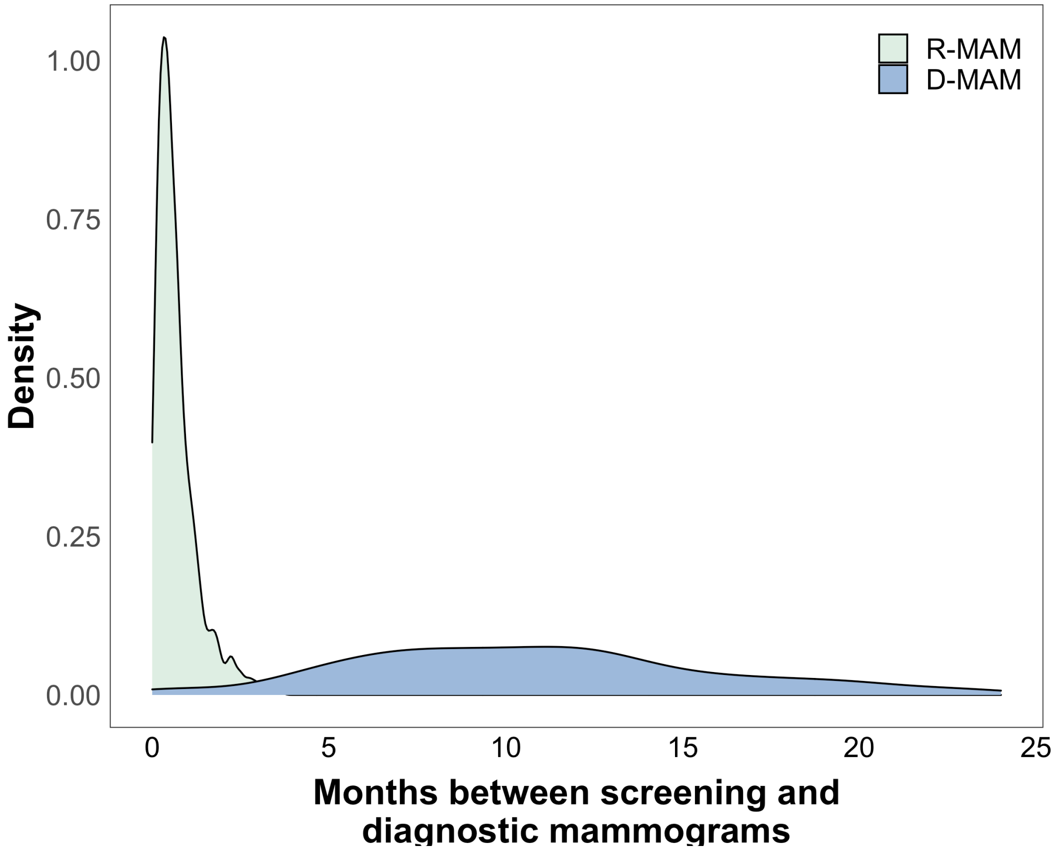

Supplementary Figure 4. Distribution of time between a screening mammogram and subsequent diagnostic mammogram based on proximity of screening to the date of breast cancer diagnosis. R-MAMs represent breast cancers diagnosed less than 4 months after a screening mammogram, while D-MAMs represent breast cancers diagnosed 4-24 months after a screening mammogram.

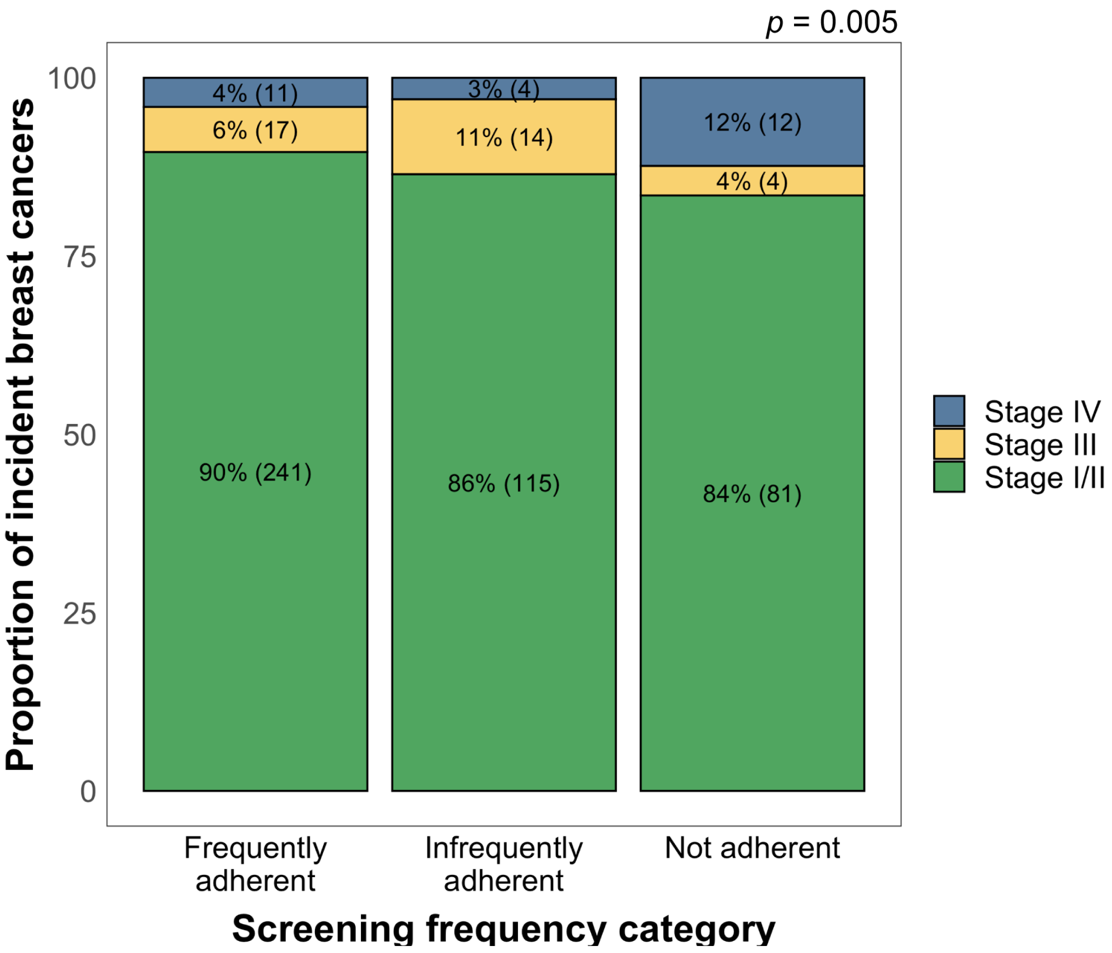

Supplementary Figure 5. Breast cancer stage distribution by frequency of adherence to breast cancer screening recommendation. P-value is from Pearson chi-square test for independence.

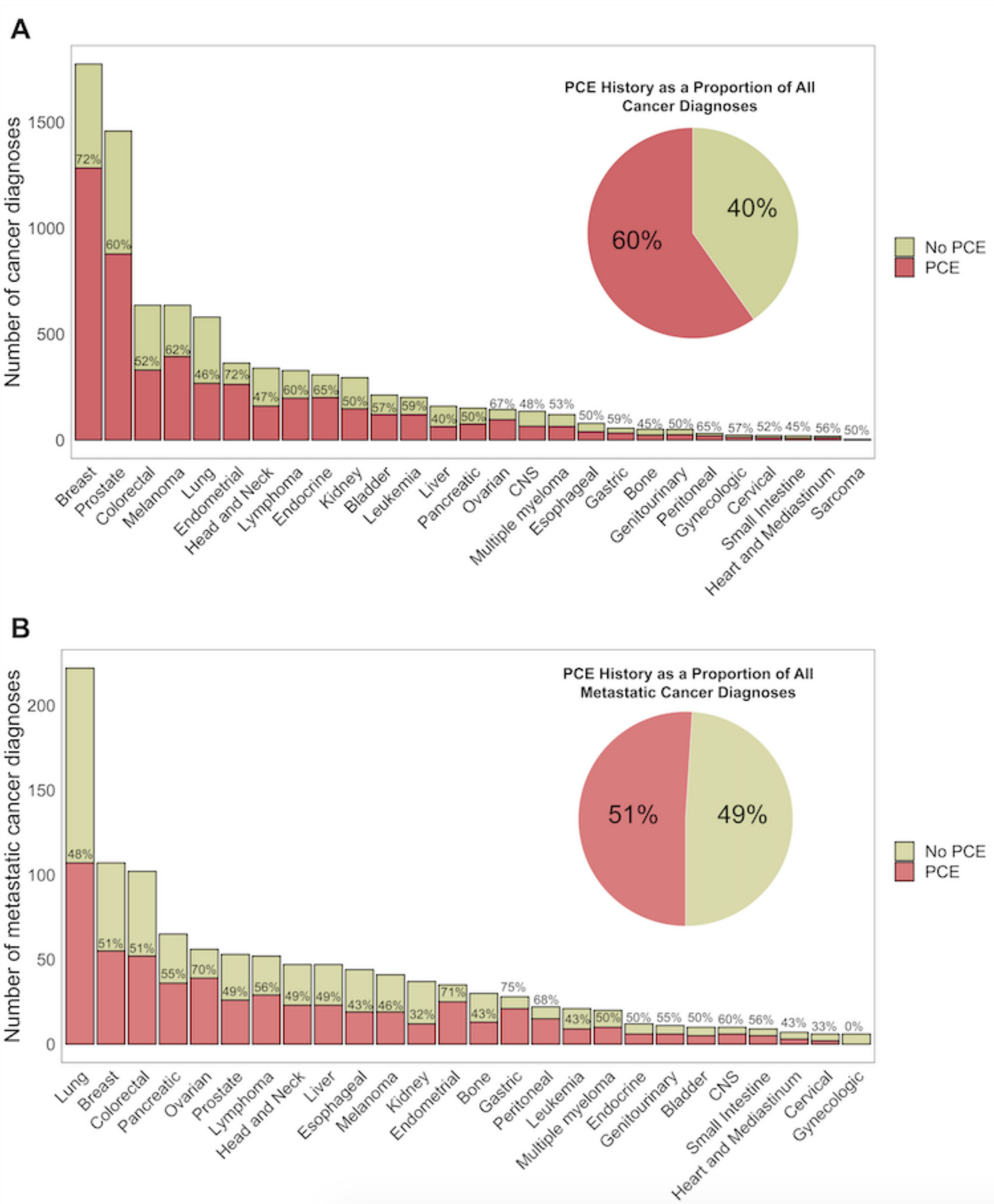

Supplementary Figure 6. Looking at patients diagnosed with (**A**) all cancer types and stages, and (**B**) specifically metastatic cancer, the proportion of patients with a history of a PCE within 2 years of diagnosis.

References (Supplementary Material)

1. Khushalani JS, Ekwueme DU, Richards TB, Sabatino SA, Guy GP, Zhang Y, Tangka F. Utilization and Cost of Mammography Screening Among Commercially Insured Women 50 to 64 Years of Age in the United States, 2012-2016. J Women's Health (2020) 29:327-337. doi:10.1089/jwh.2018.7543
2. Surveillance, Epidemiology, and End Results Program. SEER*Rx Interactive Antineoplastic Drugs Database. https://seer.cancer.gov/seertools/seerrx/ [Accessed September 17, 2020].
3. Grant SR, Walker GV, Guadagnolo BA, Koshy M, Allen PK, Mahmood U. Variation in Insurance Status by Patient Demographics and Tumor Site Among Nonelderly Adult Patients With Cancer. Cancer (2015) 121:2020-2028. doi:10.1002/cncr.29120
